# Lack of 14-3-3 proteins in *Saccharomyces cerevisiae* results in cell-to-cell heterogeneity in the expression of Pho4-regulated genes *SPL2* and *PHO84*

**DOI:** 10.1186/s12864-017-4105-8

**Published:** 2017-09-06

**Authors:** Janneke H.M. Teunissen, Marjolein E. Crooijmans, Pepijn P.P. Teunisse, G. Paul H. van Heusden

**Affiliations:** Institute of Biology, Leiden University, Sylviusweg 72, NL-2333BE Leiden, the Netherlands

**Keywords:** *Saccharomyces cerevisiae*, 14-3-3 proteins, *SPL2*, *PHO84*, *PHO* regulon, Gene expression, Potassium starvation

## Abstract

**Background:**

Ion homeostasis is an essential property of living organisms. The yeast *Saccharomyces cerevisiae* is an ideal model organism to investigate ion homeostasis at all levels. In this yeast genes involved in high-affinity phosphate uptake (*PHO* genes) are strongly induced during both phosphate and potassium starvation, indicating a link between phosphate and potassium homeostasis. However, the signal transduction processes involved are not completely understood. As 14-3-3 proteins are key regulators of signal transduction processes, we investigated the effect of deletion of the 14-3-3 genes *BMH1* or *BMH2* on gene expression during potassium starvation and focused especially on the expression of genes involved in phosphate uptake.

**Results:**

Genome-wide analysis of the effect of disruption of either *BMH1* or *BMH2* revealed that the mRNA levels of the *PHO* genes *PHO84* and *SPL2* are greatly reduced in the mutant strains compared to the levels in wild type strains. This was especially apparent at standard potassium and phosphate concentrations. Furthermore the promoter of these genes is less active after deletion of *BMH1*. Microscopic and flow cytometric analysis of cells with GFP-tagged *SPL2* showed that disruption of *BMH1* resulted in two populations of genetically identical cells, cells expressing the protein and the majority of cells with no detectible expression. Heterogeneity was also observed for the expression of GFP under control of the *PHO84* promoter. Upon deletion of *PHO80* encoding a regulator of the transcription factor Pho4, the effect of the *BMH1* deletion on *SPL2* and *PHO84* promoter was lost, suggesting that the *BMH1* deletion mainly influences processes upstream of the Pho4 transcription factor.

**Conclusion:**

Our data indicate that that yeast cells can be in either of two states, expressing or not expressing genes required for high-affinity phosphate uptake and that 14-3-3 proteins are involved in the process(es) that establish the activation state of the *PHO* regulon.

**Electronic supplementary material:**

The online version of this article (10.1186/s12864-017-4105-8) contains supplementary material, which is available to authorized users.

## Background

Ion homeostasis is an essential property of living organisms. The intracellular concentrations of ions must be tightly regulated because ions like H^+^, K^+^, Fe^2+^, Ca^2+^ and PO_4_^2−^ affect important processes and activities in many cellular systems. On the other hand, high Na^+^ concentrations are toxic for cells. Deficiencies in cation homeostasis are linked to many diseases, such as Alzheimer’s disease [1] and epilepsy [2]. Properties of ion homeostasis in plants determine their ability to grow in environments with very low or high concentrations of salts and nutrients. However, ion homeostasis is only partly understood. The yeast *Saccharomyces cerevisiae* is an excellent model organism to study ion homeostasis (for reviews see [3–5]). In this yeast the intracellular potassium concentration is relatively high at 200–300 mM, in contrast, the intracellular Na^+^ concentration is low, around 20 mM. Recently, the effect of potassium starvation has been studied at a transcriptional level [6, 7]. We identified 105 genes of which the RNA levels were significantly (*P* < 0.01) up-regulated more than 2.0-fold and 172 genes of which the mRNA levels were significantly down-regulated more than 2.0-fold [7]. It was found that several genes involved in phosphate metabolism were up-regulated during potassium starvation, indicating a link between potassium homeostasis and phosphate metabolism [7, 8].

Intracellular phosphate levels are maintained by an interplay between low- and high-affinity phosphate transporters (for review see: [9, 10]). At high phosphate levels, the low affinity phosphate transporters Pho87 and Pho90 are responsible for phosphate uptake. Under these conditions the Pho4 transcription factor is inactive following phosphorylation by the cyclin – cyclin-dependent kinase complex Pho80 – Pho85 [11, 12]. Upon phosphate shortage the Pho80 – Pho85 complex is inactivated, the Pho4 transcription factor becomes de-phosphorylated and enters the nucleus resulting in expression of a number of genes involved in phosphate uptake (*PHO* genes) [13]. These genes include among others *PHO5*, encoding an extracellular phosphatase [14], *PHO84*, encoding a high affinity phosphate transporter [15] and *SPL2*, encoding a protein with similarity to cyclin-dependent kinase inhibitors which down-regulates low-affinity phosphate transporters [16, 17]. Evidence has been provided that during potassium starvation *PHO* genes are activated by a similar mechanism as during phosphate starvation and that the entire *PHO* signaling pathway is required for regulation of *PHO84* expression [8].

14-3-3 proteins are regulatory proteins identified in all eukaryotic organisms often in multiple isoforms capable of binding to hundreds of phosphorylated proteins (for review see: [18–21]). The yeast *S. cerevisiae* has two genes encoding 14-3-3 proteins, *BMH1* and *BMH2* [22–25]. As 14-3-3 proteins participate in many signal transduction processes it is likely that they also have a role in the regulation of the *PHO* genes. It has been hypothesized that physiological changes in phosphate concentrations can modulate the affinity and specificity of interaction of 14-3-3 with its multiple targets [26]. This may implicate a role of 14-3-3 proteins in phosphate sensing mechanisms and thus in the regulation of the *PHO* genes. To further understand the control of the expression of *SPL2* and *PHO84* and the possible involvement of 14-3-3 proteins in a follow-up to our previous study [7] we investigated the effect of deletion of *BMH1* or *BMH2* on the transcriptional response to potassium starvation. These experiments revealed a very low expression of the *PHO* genes *PHO84* and *SPL2* at standard phosphate and potassium concentrations, indicating that 14-3-3 proteins are indeed involved in the regulation of *PHO* genes. We further present evidence that deletion of *BMH1* results in heterogeneity in expression of *PHO* genes in genetically identical yeast cells.

## Methods

### Strains, plasmids, primers, media and culture conditions

In this study the yeast strain BY4741 and strains derived from BY4741 were used, as listed in Table 1. Plasmids and primers used in this study are listed in Tables 2 and 3, respectively. For cultivation of yeast at defined potassium concentrations YNB medium containing very low concentrations of alkali metal cations, developed by the Translucent consortium, was used [27]. If required, histidine, leucine, methionine and/or uracil were added to a final concentration of 20 mg/L. For cultivation at defined phosphate concentrations phosphate-free YNB medium (Formedium, UK) was used. If required, potassium phosphate (pH 5.8) was added to a final concentration of 7.2 mM and potassium chloride was added to a final concentration of 50 mM. To study the effects of potassium starvation yeast strains were grown overnight at 30 °C in supplemented Translucent YNB medium containing 50 mM KCl. This culture was used to inoculate two times 50 ml of supplemented YNB medium containing 50 mM KCl yielding A_620nm_ 0.1. These cultures were grown to A_620nm_ 0.5 and cells were isolated by centrifugation. Cells from one culture were washed twice with supplemented YNB medium containing 50 mM KCl and resuspended in 50 ml supplemented YNB medium containing 50 mM KCl. Cells from the other culture were washed twice with supplemented YNB medium lacking KCl and resuspended in 50 ml supplemented YNB medium lacking KCl. Both cultures were incubated at180 rpm at 30 °C for 60 min. In a similar way the effects of phosphate starvation were studied. Yeast transformations were performed using the lithium acetate method [28].

**Table 1:** Yeast strains used in this study

Strain	Genotype	Source/Reference
BY4741	*MATa his3Δ1 leu2Δ0 met15Δ0 ura3Δ0*	Euroscarf
bmh1Δ (GG3240)	*bmh1Δ::loxP* in BY4741	This study
bmh2Δ (GG3241)	*bmh2Δ::loxP* in BY4741	This study
pho80Δ (GG3432)	*Δpho80::*KAN.MX in BY4741	This study
bmh1Δ pho80Δ (GG3433)	*bmh1Δ::loxP Δpho80::*KAN.MX in BY4741	This study
BY4741 SPL2-GFP (GG3434)	*SPL2-GFP (HIS3)* in BY4741	This study
bmh1Δ SPL2-GFP (GG3435)	*bmh1Δ::loxP SPL2-GFP (HIS3)* in BY4741	This study
bmh2Δ SPL2-GFP (GG3444)	*bmh2Δ::loxP SPL2-GFP (HIS3)* in BY4741	This study
BY4741 (pRS305) (GG3436)	*leu2Δ0*::pRS305(*LEU2*) in BY4741	This study
BY4741 (P_PHO84_-GFP) (GG3437)	*leu2Δ0*::pRS305[P_PHO84_-GFP](*LEU2*) in BY4741	This study
BY4741 (P_CYC1_-GFP) (GG3438)	*leu2Δ0*::pRS305[P_CYC1_-GFP](*LEU2*) in BY4741	This study
bmh1Δ (pRS305) (GG3439)	*bmh1Δ::loxP leu2Δ0*::pRS305(*LEU2*) in BY4741	This study
bmh1Δ (P_PHO84_-GFP) (GG3440)	*bmh1Δ::loxP leu2Δ0*::pRS305[P_PHO84_-GFP](*LEU2*) in BY4741	This study
bmh1Δ (P_CYC1_-GFP) (GG3441)	*bmh1Δ::loxP leu2Δ0*::pRS305[P_CYC1_-GFP](*LEU2*) in BY4741	This study
BY4741 (pRS305[PHO4]) (GG3442)	*leu2Δ0*::pRS305[PHO4](*LEU2*) in BY4741	This study
bmh1Δ (pRS305[PHO4]) (GG3443)	*bmh1Δ::loxP leu2Δ0*::pRS305[PHO4](*LEU2*) in BY4741	This study

**Table 2:** Plasmids

Plasmid	Properties	Source/Reference
pRS316	Yeast centromeric plasmid. *URA3* marker.	[52]
pRS316[P_PHO84_-GFP-T_PHO84_] (pRUL1336)	pRS316 containing the *PHO84* promoter (600 bp), GFP and the *PHO84* terminator (436 bp)	This study
pRS316[P_CYC1_-GFP-T_CYC1_] (pRUL1339)	pRS316 containing the *CYC1* promoter (300 bp), GFP and the *CYC1* terminator (220 bp)	This study
pRS316[P_SPL2_-GFP-T_SPL2_] (pRUL1354)	pRS316 containing the *SPL2* promoter (647 bp), GFP and the *SPL2* terminator (324 bp)	This study
pUG6	Plasmid containing the KAN.MX casette for gene disruptions	[53]
pUG36	Centromeric plasmid to make N-terminal GFP fusions.	Güldener and Hegemann, unpublished
pRS305	Yeast integration plasmid. *LEU2* marker.	[52]
pRS305[P_PHO84_-GFP-T_PHO84_] (pRUL1359)	pRS305 containing the *PHO84* promoter (600 bp), GFP and the *PHO84* terminator (436 bp)	This study
pRS305[P_CYC1_-GFP-T_CYC1_] (pRUL1360)	pRS305 containing the *CYC1* promoter (300 bp), GFP and the *CYC1* terminator (220 bp)	This study
pRS313	Yeast centromeric plasmid. *HIS3* selection marker.	[52]
pRS313[PHO4](pRUL1334)	pRS313 containing *PHO4*	[7]
pRS313[BMH1] (pRUL1330)	pRS313 containing a 3.2 kb genomic DNA fragment with *BMH1*	I.G. Anemaet, unpublished results
pRS305[PHO4] (pRUL1358)	pRS305 containing *PHO4*	This study
YCplac33	Yeast centromeric plasmid. *URA3* marker	[54]
YCplac33[BMH1]	YCplac33 with a 3.2 kb genomic DNA fragment with *BMH1*	[55]

**Table 3:** Primers

Primer	Sequence (5′- 3′)
Bmh1-kanMX- Fw	GCAAGTGAGAAGAAAAAGCAAGTTAAAGATAAACTAAAGATAAAACAGCTGAAGCTTCGTACGC
Bmh1-kanMX-Rv	AGATTTATCAGAATACTTACTTTGGTGCTTCACCTTCGGCGGCAGCGCATAGGCCACTAGTGGATCTG
Bmh2-kanMX-Fw	GAAAAATTATCAAATCAACAAAAAGTACCCGTTACAACAAAAAAACAGCTGAAGCTTCGTACGC
Bmh2-kanMX-Rv	GCAAGAAAACTGGAGTGGTAAATCTTCATTTCCCCTTGTATTTCTGCATAGGCCACTAGTGGATCTG
PHO84-qPCR-Fw	ACAACCTTG TTGATCCCAG AA
PHO84-qPCR-Rv	TGCTTCATGTTGAAGTTGAGATG
SPL2-qPCR-Fw	CCGAGGAGATCGCTTCTCTA
SPL2-qPCR-Rv	ACGCTGCGCTCTACTTGAAT
ACT1-qPCR-Fw	CTGCCGGTATTGACCAAACT
ACT1-qPCR-Rv	CGGTGATTTCCTTTTGCATT
PHO80-kanMX-Fw	AAGCTATCATAAGACGAGGATATCCTTTGGAGACTCATAGAAATCCAGCTGAAGCTTCGTACGC
PHO80-kanMX-Rv	TTTTGCTCAATCATGATTGCTTTCATAATACCCCACGAAAAATCACCGCGGCCGCATAGGCCAC
SPL2-GFP-Fw2	ATTGACGAAGACATATTCGAAGATTCGTCTGACGAAGAACAATCACGTACGCTGCAGGTCGAC
SPL2-GFP-Rv	GTCAATGCATATGTAACAGTACAGAGGTAGAAGGTATGTGTATCGATGAATTCGAGCTCG
P-pho84-Fw	AAA GAGCTC AATCAGTATT ACGCACGTTGGT
P-pho84-Rv	AAA ACTAGT CATTTGGATTGTATTCGTGGAGT
T-pho84-Fw	AAA GGATCC TAAAAGCCT CAAAGATGCA CTAA
T-pho84-Rv	AAA GAATTC CTGTCCCACAGGTGCCATTG
P-cyc1-Fw	AAA GAGCTC GTTCATTTGG CGAGCGTTGG
P-cyc1-Rv	AAA ACTAGT CATTATTAATTTAGTGTGTGTATTTG
T-cyc1-Fw	AAA GGATCC TAA ACAGGCCCCT TTTCCTTTGTC
T-cyc1-Rv	AAA GAATTC ATGTTACATGCGTACACGCGT
P-spl2-Fw	AAA GAGCTC TTTACACTGGGATATTACAAGAC C
P-spl2-Rv2	AAA ACTAGT CATCTGTCCAATTTGCCCCTG
T-spl2-Fw	AAA GGATCC TGATTGCATCTCTTAATCGTTACAC
T-spl2-Rv	AAA GAATTC AAAGGGCCAGCGAATGCGCG
GFP-Fw	AAA ACTAGT ATGTCTAAAGGTGAAGAATTATTCA
GFP-Rv	AAA GGATCC TTTGTACAATTCATCCATACCATA
PHO4-Fw	GG GAATTC GTCTCTGTCTAATGCGGTCAC
PHO4-Rv	GG GGATCC GTTCTCTCAAATCTTCCAACTGATC

### Construction of yeast strains

For disruption of *BMH1* and *BMH2* a DNA fragment was generated by PCR on plasmid pUG6 using the primer combinations Bmh1-kanMX-Fw – Bmh1-kanMX-Rv and Bmh2-kanMX- Fw -- Bmh2-pUG6-Rv, respectively. These DNA fragments were used to transform BY4741 and transformants were selected on YPD plates containing 150 μg/ml G418. Correct integration was verified by PCR. The KAN.MX fragment was removed after introduction of pNatCre [29]. For disruption of *PHO80* a DNA fragment was generated by PCR on plasmid pUG6 using the primer combinations PHO80-kanMX- Fw – PHO80-kanMX- Rv. This DNA fragment was used to transform BY4741 and bmh1∆ and transformants were selected on YPD plates containing 150 μg/ml G418. Correct integration was verified by PCR.

To tag chromosomal *SPL2* at its 3′-end with GFP a PCR fragment was generated using the primer combination SPL2-GFP-Fw2 - SPL2-GFP-Rv and plasmid pYM28 [30] as template. This fragment was used to transform BY4741, bmh1∆ and bmh2Δ yielding the histidine prototrophic strains BY4741 SPL2-GFP, bmh1∆ SPL2-GFP and bmh2∆ SPL2-GFP, respectively. Correct integration was verified by PCR.

For integration of pRS305 or plasmids derived from pRS305 BY4741 or bmh1∆ were transformed with these plasmids and leucine prototrophic transformants were selected. Yeast strains carrying plasmids were obtained by transforming parental strains with the appropriate plasmids followed by selection for uracil prototrophy.

### Construction of plasmids

Reporter plasmids to analyze promoter activity were generated from pRS316. A fragment with the GFP coding sequences and restriction sites for *Spe*I and *BamH*I at the ends was generated by PCR using the primer combination GFP-Fw -- GFP-Rv and pUG36 as template. This fragment was ligated into pRS316 after digestion with *Spe*I and *Bam*HI. DNA fragments with restriction sites for *Sac*I and *Spe*I at the ends containing sequences of the promoter of *PHO84*, *SPL2* and *CYC1* were obtained by PCR on genomic BY4741 DNA using the primer combinations P-pho84-Fw -- P-pho84-Rv, P-spl2-Fw -- P-spl2-Rv2 and P-cyc1-Fw -- P-spl2-Fw, respectively. DNA fragments with restriction sites for *Bam*HI and *Eco*RI at the ends containing sequences of the transcription terminator of *PHO84*, *SPL2* and *CYC1* were obtained by PCR on genomic BY4741 DNA using the primer combinations T-pho84-Fw -- T-pho84-Rv, T-spl2-Fw -- T-spl2-Rv2 and T-cyc1-Fw -- T-cyc1-Fw, respectively. The promoter fragments were ligated in pRS316 containing GFP using the restriction enzymes *Sac*I and *Spe*I, whereas terminator fragments were ligated after digestion with *Bam*HI and *Eco*RI, yielding pRS316[P_PHO84_-GFP-T_PHO84_], pRS316[P_SPL2_-GFP-T_SPL2_] and pRS316[P_CYC1_-GFP-T_CYC1_]. The position of the start codon of *SPL2* is unclear. The coding region of *SPL2* is annotated between coordinates 375,100 and 374,654 of chromosome VIII (SGD, www.yeastgenome.org). Ten bp upstream of the annotated start codon an out of frame ATG sequence exists making the annotated start codon less likely to be the genuine start codon. Therefore a more likely start codon is located 85 bp downstream of the annotated start codon. This problem has been mentioned before (supplementary data in: [31]). For our promoter constructs we considered this downstream ATG as the start codon.

pRS305[P_PHO84_-GFP-T_PHO84_] and pRS305[P_CYC1_-GFP-T_CYC1_] were made by transferring the *Sac*I – *Hind*III fragments containing the promoter, GFP and terminator from pRS316[P_PHO84_-GFP-T_PHO84_] and pRS316[P_CYC1_-GFP-T_CYC1_], respectively, to pRS305. pRS305[PHO4] was prepared by transferring a *Bam*HI – *Sal*I fragment with *PHO4* from pRS313[PHO4] to pRS305.

### Transcriptome analysis by SAGE-tag sequencing and qRT-PCR

Cultivation of BY4741, bmh1∆ and bmh2∆, isolation of RNA and SAGE-tag sequencing was done as described previously [7]. At least 1.0 million matching reads per sample were obtained by SAGE-tag sequencing. qRT-PCR was performed as described earlier [7]. To measure transcript levels of *PHO84* and *SPL2* primer combinations PHO84-qPCR-Fw -- PHO84-qPCR-Rv and SPL2-qPCR-Fw -- SPL2-qPCR-Rv, respectively, were used. Transcript levels were normalized against expression of *ACT1*, measured using the primer combination ACT1-qPCR-Fw -- ACT1-qPCR-Rv.

### Confocal microscopy and flow cytometry

Yeast cells were grown in potassium- or phosphate-free YNB medium supplemented with KCl, potassium phosphate, histidine, methionine, uracil and leucine, when required. For image acquisition a Zeiss LSM 5 Exciter-AxioImager M1 confocal microscope with a Plan-Aprochromat objective (63X/1.4 Oil DIC) and Zeiss ZEN 2009 software were used. GFP was imaged with excitation at 488 nm and emission at 505–530 nm. Adobe Photoshop software was used to increase the visibility of the GFP signals and cells by linear adjustments of intensities. For flow cytometry, a Merck-Millipore Guava EasyCyte 5 Flow Cytometer was used. Fluorescence was determined after excitation at 488 nm and using the standard green 525/30 nm emission filter. For each analysis 5000 cells were used.

## Results

### Levels of *PHO84* and *SPL2* RNA are strongly reduced in *bmh* mutants

Our previous study showed that potassium starvation resulted in a strong induction of genes like *PHO84* and *SPL2* known to be activated at low phosphate conditions. In order to address the role of 14-3-3 proteins in the expression of *PHO* genes we analyzed the effect of potassium starvation on the genome-wide transcript profiles of *bmh1*∆ and *bmh2*∆ deletion strains. To this end these strains were grown in potassium-free YNB medium supplemented with 50 mM KCl [27]. Exponentially growing cells were transferred to medium containing 50 mM KCl or lacking KCl and grown for 60 min. RNA was isolated for transcriptome analysis by Serial Analysis of Gene Expression (SAGE)-tag sequencing. The complete dataset is given in Additional file 1. Although the variation in expression between the different replicates was larger than usual, the data did show a strong reduction in the level of *PHO84* and *SPL2* RNA in the *bmh1*∆ and *bmh2*∆ deletion strains (Table 4). This effect is most apparent at standard potassium concentrations (50 mM KCl). Other *PHO* genes like *VTC2, GDE1,VTC1, VTC3, PHO89*, *PHO8* and *PHM6* were affected as well by the *bmh1* deletion, but these differences were not significant (Additional file 2). Few other genes were found to be affected by the *BMH1* or *BMH2* deletion. The RNA levels of 10 genes were significantly (*P* < 0.01) increased or decreased more than 2.0-fold in the *bmh1∆* mutant after growth at standard potassium concentration. In the *bmh2*∆ strain the RNA level of 6 genes were significantly affected (Additional file 3). A different set of genes was found for the *bmh1* and *bmh2* deletions, but for some genes the effect was apparent in both mutants, although not significant. The effect of the *bmh1* deletion on *PHO84* and *SPL2* could be confirmed by qRT-PCR (Table 5). The effect of the *bmh1* deletion can be complemented by introduction of a wild type *BMH1* allele on a centromeric plasmid (YCplac33[BMH1]) (Table 5). Introduction of this plasmid in a wild type strain resulted in up-regulation of the *SPL2* and *PHO84* expression in line with a role of *BMH1* in the regulation of these genes (Table 5).

**Table 4:** Effect of *bmh1* and *bmh2* deletion on RNA levels of *PHO84* and *SPL2* (SAGE-tag sequencing)

	RNA level (reads per million) (± SD)					
Gene	BY4741		bmh1Δ		bmh2Δ	
	50 mM (*n* = 4)	0 mM (*n* = 4)	50 mM (*n* = 3)	0 mM (*n* = 3)	50 mM (*n* = 3)	0 mM (*n* = 3)
*PHO84*	50 ± 27	516 ± 214	2 ± 1^a^	243 ± 140	6 ± 0.5^c^	235 ± 16
*SPL2*	31 ± 5	224 ± 69	3 ± 0^b^	95 ± 33	7 ± 1^d^	163 ± 26

^a^Student’s t-test indicated a significant difference between bmh1Δ and BY4741 at 50 mM KCl (*P* = 0.03)

^b^Student’s t-test indicated a significant difference between bmh1Δ and BY4741 at 50 mM KCl (*P* = 0.0002)

^c^Student’s t-test indicated a significant difference between bmh2Δ and BY4741 at 50 mM KCl (*P* = 0.04)

^d^Student’s t-test indicated a significant difference between bmh2Δ and BY4741 at 50 mM KCl (*P* = 0.004)

**Table 5:** RNA levels of *PHO84* and *SPL2* determined by qRT-PCR and complementation by wild type *BMH1*

	RNA level (arbitrary units) (± SD; *n* = 3)			
Strain	*PHO84*		*SPL2*	
	50 mM	0 mM	50 mM	0 mM
BY4741 pRS313^a^	0.18 ± 0.03	1.10 ± 0.20	0.24 ± 0.12	1.50 ± 0.34
bmh1Δ pRS313	0.03 ± 0.01^b^	0.98 ± 0.31	0.05 ± 0.02	1.44 ± 0.21
BY4741 pRS313[BMH1]	1.01 ± 0.01	1.23 ± 0.40	1.05 ± 0.19	2.03 ± 0.22
bmh1Δ pRS313[BMH1]	0.18 ± 0.12	0.78 ± 0.23	0.33 ± 0.16	1.34 ± 0.11

^a^pRS313, empty plasmid control

^b^Student’s t-test indicated a significant difference between bmh1Δ and BY4741 at 50 mM KCl (*P* = 0.01)

### Effect of *bmh1* deletion on the *PHO84* and *SPL2* promoter

RNA levels are influenced by both transcription and degradation. Therefore, we investigated the effect of 14-3-3 deletion on the activity of the *PHO84* and *SPL2* promoter. As the effect of the *BMH1* deletion is stronger than that of the *BMH2* deletion we mainly focused on the former deletion. To this end, we made reporter constructs in the centromeric pRS316 plasmid by inserting GFP under control of *PHO84* or *SPL2* promoter sequences or *CYC1* promoter sequences as a control. Expression of *CYC1*, encoding isoform 1 of Cytochrome c, is not significantly affected by potassium starvation [7]. These reporter plasmids were introduced in wild type and *bmh1*∆ deletion strains and GFP expression was determined after growth at 50 and 0 mM KCl by flow cytometry. As shown in Table 6 the *PHO84* promoter has an approx. 2-fold lower activity in the *bmh1*∆ mutant, whereas the *SPL2* promoter is more than 4-fold less active. These results indicate that the lower levels of *PHO84* and *SPL2* RNA are at least partly caused by a lower transcription. The increase in RNA levels of *PHO84* and *SPL2* (Tables 4 and 5) upon potassium starvation is also reflected in the activation of the promoters during potassium starvation (Table 6).

**Table 6:** Activity of the *PHO84*, *SPL2* and *CYC1* promoter determined by flow cytometry using GFP reporters

	GFP fluorescence (arbitrary units) (± SD; *n* = 3)	
Strain	50 mM	0 mM
Experiments *PHO84*		
BY4741 P_PHO84_– GFP–T_PHO84_	30 ± 1	58 ± 2
BY4741 P_CYC1_ – GFP–T_CYC1_	198 ± 29	179 ± 8
bmh1Δ P_PHO84_– GFP–T_PHO84_	13 ± 2^a^	37 ± 3
bmh1Δ P_CYC1_ – GFP–T_CYC1_	204 ± 12	146 ± 54
Experiments *SPL2*		
BY4741 P_SPL2_– GFP–T_SPl2_	3.7 ± 1.6	7.1 ± 2.8
BY4741 P_CYC1_ – GFP–T_CYC1_	204 ± 12	219 ± 30
bmh1Δ P_SPL2_– GFP–T_SPL2_	0.8 ± 0.3^b^	2.7 ± 0.5
bmh1Δ P_CYC1_ – GFP–T_CYC1_	131 ± 5	139 ± 24

^a^Student’s t-test indicated a significant difference between bmh1Δ and BY4741 at 50 mM KCl (*P* < 0.001)

^b^Student’s t-test indicated a significant difference between bmh1Δ and BY4741 at 50 mM KCl (*P* = 0.03)

*PHO84* and *SPL2* are regulated by the transcription factor Pho4 [32]. After introduction of our reporter construct for the *PHO84* promoter in a *pho4*∆ deletion strain hardly any GFP fluorescence could be detected (data not shown), confirming the importance of the Pho4 transcription factor for the expression of *PHO84*. When phosphate is available Pho4 is de-activated by phosphorylation by the Pho80 – Pho85 complex. Deletion of *PHO80* results in activation of the *PHO* genes. To investigate the effect of *PHO80* disruption on the activity of the *PHO84* and *SPL2* promoters we introduced the reporter constructs for these promoters into wild type and *bmh1*∆ cells with an additional deletion of *PHO80* and determined GFP fluorescence after growth in the presence of 50 mM KCl. As shown in Table 7, both the *PHO84* and *SPL2* promoters are highly active in the *pho80*∆ mutant. In the *bmh1*∆ *pho80*∆ double mutant the negative effect of the *bmh1* deletion is lost. These data suggest that Bmh1 affects *PHO84* and *SPL2* promoter activity upstream of the Pho4 transcription factor.

**Table 7:** Effect of *pho80* deletion on the activity of the *PHO84*, *SPL2* and *CYC1* promoter

Strain	GFP fluorescence (arbitrary units) (± SD; *n* = 3)		
	*PHO84*	*SPL2*	*CYC1*
BY4741	60 ± 26	5.7 ± 0.9	249 ± 38
bmh1Δ	18 ± 0.8	0.5 ± 0.3	219 ± 31
pho80Δ	1128 ± 176	118 ± 17	155 ± 12
bmh1∆pho80Δ	1528 ± 279	147 ± 23	332 ± 117

### *bmh1* deletion results in heterogenic expression of Spl2-GFP

**Fig. 1:**
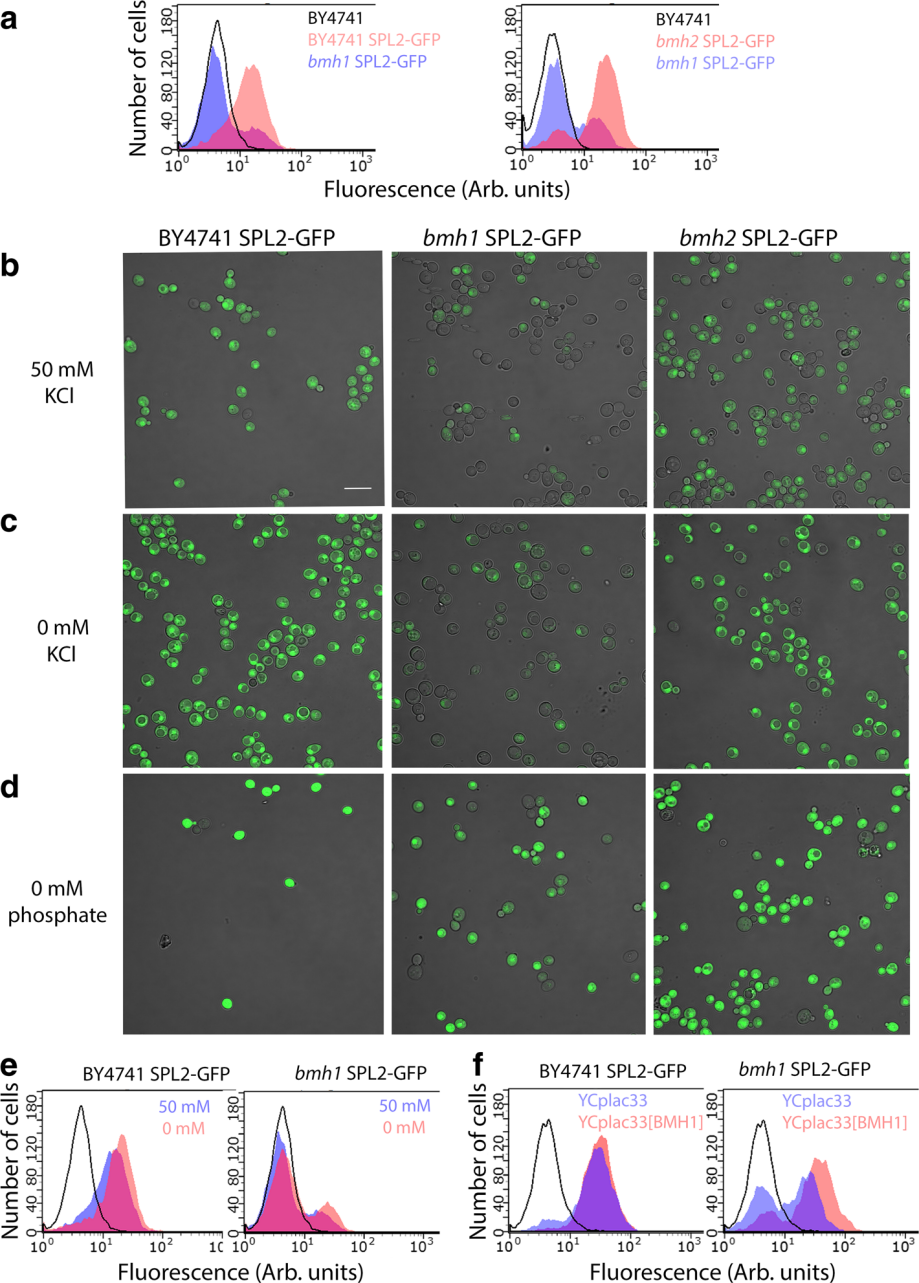
Heterogenic expression of SPL2-GFP in bmh1∆ cells. **a** left panel, flow cytometry of BY4741 cells (line, no fill), BY4741 SPL2-GFP cells (red) and bmh1Δ SPL2-GFP cells (blue) grown in YNB medium with 50 mM KCl; right panel, flow cytometry of BY4741 cells (line, no fill), bmh2Δ SPL2-GFP cells (red) and bmh1∆ SPL2-GFP cells (blue) grown in YNB medium with 50 mM KCl. **b** confocal microscopy of BY4741 SPL2-GFP, bmh1Δ SPL2-GFP and bmh2Δ SPL2-GFP cells grown in YNB medium with 50 mM KCl. Scale bar 10 μm. **c** confocal microscopy of BY4741 SPL2-GFP, bmh1Δ SPL2-GFP and bmh2Δ SPL2-GFP cells grown for 2 h in YNB medium without KCl. **d** confocal microscopy of BY4741 SPL2-GFP, bmh1Δ SPL2-GFP and bmh2Δ SPL2-GFP cells grown for 2 h in YNB medium without phosphate. **e** left panel: flow cytometry of BY4741 cells (line, no fill), BY4741 SPL2-GFP cells grown in YNB medium with 50 mM KCl (blue) or without KCl (red). Right panel: flow cytometry of BY4741 cells (line, no fill), bmh1Δ SPL2-GFP cells grown in YNB medium with 50 mM KCl (blue) or without KCl (red). **f** left panel: flow cytometry of BY4741 cells (line, no fill), BY4741 SPL2-GFP cells containing YCplac33 (blue) and BY4741 SPL2-GFP cells containing YCplac33[BMH1] (red) grown in YNB medium with 50 mM KCl. Right panel flow cytometry of BY4741 cells (line, no fill), bmh1Δ SPL2-GFP cells containing YCplac33 (blue) and bmh1Δ SPL2-GFP cells containing YCplac33[BMH1](red) grown in YNB medium with 50 mM KCl

To analyze the effect of the *bmh1* deletion on the levels and localization of Spl2 at the protein level, we made a C-terminal fusion with GFP by integrating sequences encoding GFP at the 3′-end of the *SPL2* coding region in the wild type, *bmh1*∆ and *bmh2*∆ strains. Determination of GFP levels by flow cytometry in cells grown at 50 mM KCl revealed two populations of cells of the *bmh1*∆ mutant, one population expressing GFP, the other (larger) population not expressing Spl2-GFP (Fig. 1a, left panel). Analysis of the cells by confocal microscopy confirmed that the majority of *bmh1*∆ cells has no expression of Spl2-GFP, whereas a relatively small number of cells has clear Spl2-GFP expression (Fig. 1b). Even by using a high laser power during microscopy Spl2-GFP expression could not be detected in the majority of cells (data not shown). Analysis by flow cytometry of bmh2∆ SPL2-GFP cells showed that a small fraction of the cells lack expression of SPL2-GFP (Fig. 1a, right panel). Similar results were obtained by confocal microscopy (Fig. 1b). The heterogeneity found for bmh1∆ SPL2-GFP cells cannot be explained by a cell cycle-dependent expression of *SPL2* as in both populations single cells, small budded cells and large budded cells are present. Most of the wild type cells expresses Spl2-GFP, only in a few cells Spl2-GFP cannot be detected (Fig. 1b). Similar results were obtained when instead of GFP cyano fluorescent protein (CFP) was used to tag Spl2 (in a typical experiment 84% of the wild type cells showed expression of SPL2-CFP and 42% of the *bmh1*∆ cells showed expression of SPL2-CFP). Heterogenic expression of *SPL2* is consistent with the lower levels of *SPL2* RNA and lower activity of the *SPL2* promoter in the total population of cells. Analysis of dividing cells revealed that almost 100% of the large budded cells has the same expression level in both the mother and the daughter cell after growth at 50 mM KCl (data not shown). This indicates that the expression state is relatively stable.

To address the question whether *bmh1*∆ cells that do not express Spl2-GFP at standard potassium concentrations are able to express SPL2-GFP in the absence of potassium, we cultivated wild type, *bmh1*∆ SPL2-GFP and *bmh2*∆ SPL2-GFP cells at 0 mM KCl. As shown in Fig. 1(c and e) both confocal microscopy and flow cytometry showed that still two populations of cells exist, one population with high expression of Spl2-GFP, the other population with low expression. However, the latter population has a clearly detectable expression, indicating that this population of cells is still capable of expression of SPL2-GFP. It is of interest that the localization of Spl2-GFP is changed upon potassium starvation both in wild type and mutant cells (Fig. 1c). This change in localization was not observed for free GFP expressed under control of the SPL2 promoter (Additional file 4). Cultivation in the absence of phosphate resulted in an even further increased expression of SPL2-GFP in both wild type and mutant strains (Fig. 1d). Introduction of a wild type copy of *BMH1* to *bmh1* SPL2-GFP cells restored the expression of SPL2-GFP to nearly the same level as in BY4741 SPL2-GFP cells, although there are still cells present with no expression, maybe caused by plasmid loss (Fig. 1f).

### Heterogenic expression of *PHO84*

**Fig. 2:**
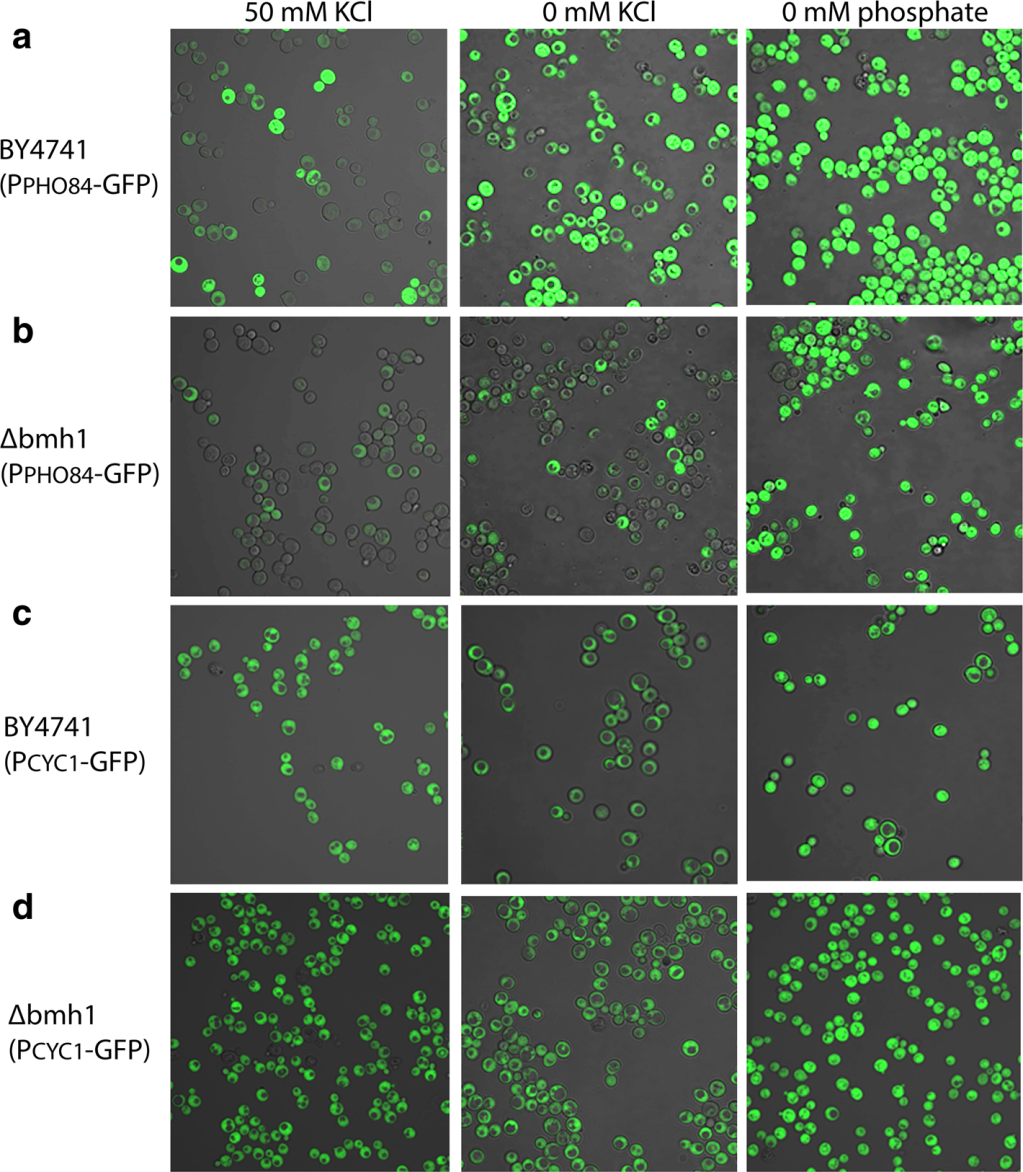
Effect of *bmh1* deletion on the *PHO84* promoter and activation by low potassium or phosphate. **a** confocal microscopy of wild type (BY4741) cells expressing GFP under control of the *PHO84* promoter (BY4741 (P_PHO84_-GFP)) after cultivation in YNB medium with 50 mM KCl, after cultivation for 1 h in YNB medium lacking potassium (0 mM KCl) and after cultivation for 1 h in YNB medium lacking phosphate (0 mM phosphate). **b** confocal microscopy of ∆*bmh1* cells expressing GFP under control of the *PHO84* promoter (∆bmh1 (P_PHO84_-GFP)) after cultivation in YNB medium with 50 mM KCl, after cultivation for 1 h in YNB medium lacking potassium (0 mM KCl) and after cultivation for 1 h in YNB medium lacking phosphate (0 mM phosphate). **c** confocal microscopy of wild type (BY4741) cells expressing GFP under control of the *CYC1* promoter (BY4741 (P_cyc1_-GFP)) after cultivation in YNB medium with 50 mM KCl, after cultivation for 1 h in YNB medium lacking potassium (0 mM KCl) and after cultivation for 1 h in YNB medium lacking phosphate (0 mM phosphate). **d** confocal microscopy of ∆bmh1 cells expressing GFP under control of the *CYC1* promoter (∆bmh1 (P_CYC1_-GFP)) after cultivation in YNB medium with 50 mM KCl, after cultivation for 1 h in YNB medium lacking potassium (0 mM KCl) and after cultivation for 1 h in YNB medium lacking phosphate (0 mM phosphate)

To investigate whether also *PHO84* has an heterogenic expression we transferred the reporter constructs for *PHO84* promoter activity mentioned above into the integrating plasmid pRS305 and integrated these constructs into the genome of wild type and *bmh1*∆ cells. Subsequently, these cells were grown under standard potassium concentrations (50 mM) and then transferred to a medium containing 50 mM KCl or to medium lacking potassium or phosphate. As shown in Fig. 2, at 50 mM KCl in wild type cells two populations of cells are found, one expressing GFP indicating an active *PHO84* promoter, the other population having a low expression of GFP, indicating a less active *PHO84* promoter. Transferring these cells to a medium lacking potassium or phosphate resulted in expression in all cells, indicative for activation of the *PHO84* promoter. At 50 mM KCl the *bmh1*∆ cells show two populations of cells, cells expressing GFP and cells with a very low expression of GFP. By using a high laser power during microscopy, in the latter population of cells GFP could be detected, indicating that in all cells the *PHO84* promoter has at least some activity. However, the expression was considerably lower in *bmh1*∆ cells than in wild type cells. Transferring to a medium without potassium or without phosphate resulted in induction of expression in all cells. However, the induction by low phosphate was much stronger than by low potassium. As a control similar experiments were performed to investigate the effect of the *bmh1*∆ deletion on the *CYC1* promoter. As shown in Fig. 2 (c and d) the *CYC1* promoter is active in all cells in media with 50 mM KCl, as well as in media lacking potassium or phosphate.

### Effect of an additional copy of *PHO4*

**Fig. 3:**
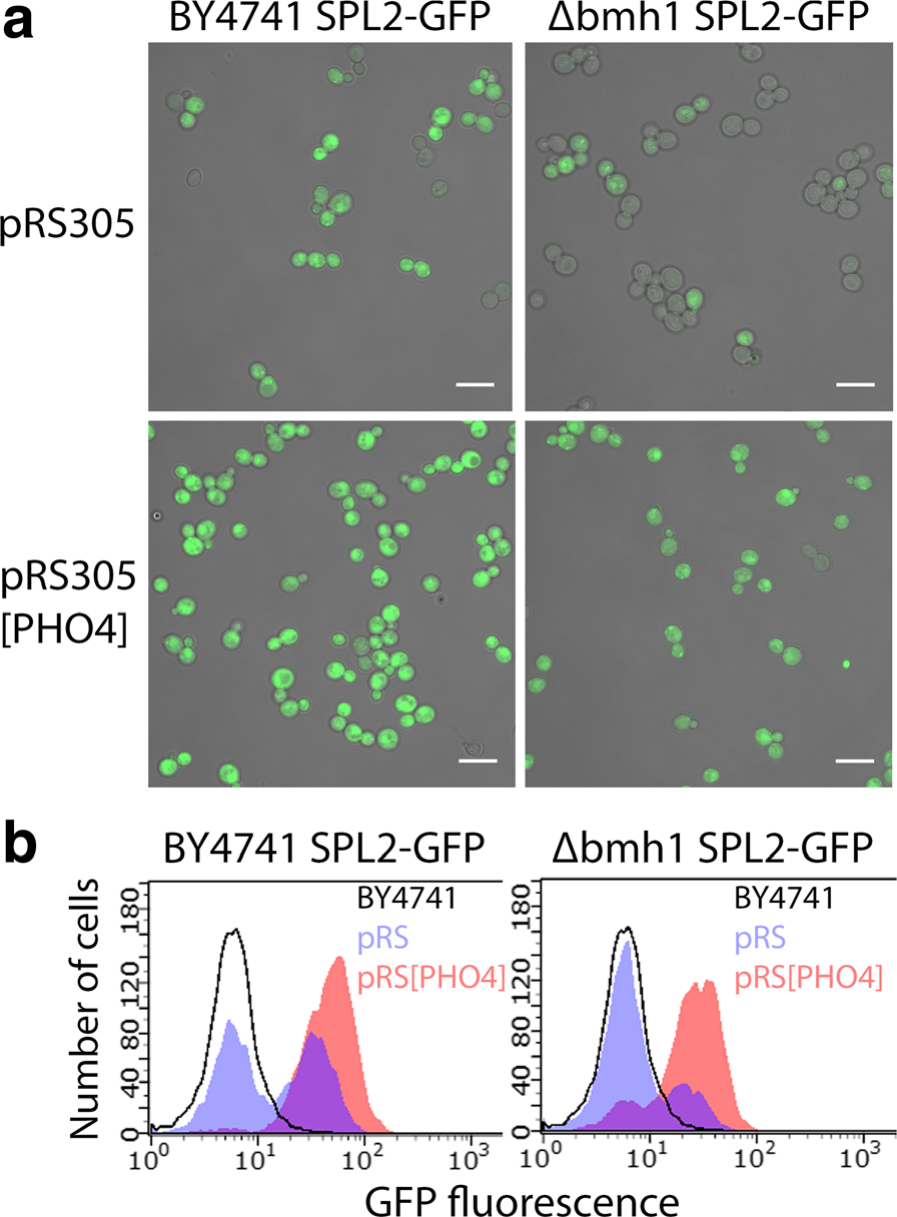
Effect of an additional copy of *PHO4* on SPL2-GFP expression. **a** Confocal microscopy of BY4741 SPL2-GFP and ∆bmh1 SPL2-GFP with integrated pRS305 or pRS305[PHO4] cells grown in YNB medium with 50 mM KCl. Scale bar 10 μm. **b** Flow cytometry of BY4741 SPL2-GFP (left panel) and ∆bmh1 SPL2-GFP (right panel) with integrated pRS305 (blue) or pRS305[PHO4] (red) cells grown in YNB medium with 50 mM KCl. Line, no fill: BY4741 cells

Induction of expression of the *PHO* genes at low phosphate concentrations requires the activation of the transcription factor Pho4. To investigate the effect of an additional copy of the *PHO4* gene on the expression of *SPL2*-GFP we inserted *PHO4* in the integrating vector pRS305 and integrated this vector into the genome of wild type and *bmh1*∆ cells expressing *SPL2*-GFP. The resulting strains were grown at 50 and 0 mM KCl and the expression of Spl2-GFP was analyzed by confocal microscopy and flow cytometry. As shown in Fig. 3 addition of an extra copy of *PHO4* resulted in an increased expression of Spl2-GFP in almost all cells and the absence of heterogeneity in expression. These data suggest that the effect of the *bmh1* deletion is not downstream of Pho4. Quantification of expression by flow cytometry showed that with the additional copy of *PHO4* the expression of SPL2-GFP in the *bmh1*∆ cells is still lower than in the BY4741 cells (BY4741 pRS305: 14.5 ± 0.7; BY4741 pRS305[PHO4]: 43 ± 3, bmh1∆ pRS305: 7.0 ± 0.3, bmh1∆ pRS305[PHO4]: 25 ± 2; arbitrary units, ± SD, *n* = 4).

## Discussion and conclusions

Upon potassium starvation mRNA levels of the *PHO84* and *SPL2* genes both related to phosphate metabolism are highly elevated [6–8]. In the present study we showed that deletion of either one of the 14-3-3 genes *BMH1* or *BMH2* resulted in decreased mRNA levels of these genes, especially apparent at standard potassium concentrations (Tables 4 and 5). These lower mRNA levels can partly be explained by a lower activity of the *PHO84* and *SPL2* promoter in the *bmh1*∆ mutant (Table 6). Further analysis at the cellular level by confocal microscopy and flow cytometry revealed that at standard phosphate and potassium concentrations in *bmh1*∆ cells the expression of *SPL2* is highly heterogenic with only a small fraction of the cells having a substantial expression of this gene (Fig. 1). This observation can explain the low levels of mRNA and the low activity of the *PH084* and *SPL2* promoters in the total population of cells (Tables 4, 5 and 6). Upon potassium or phosphate deprivation the expression of these genes is induced in all cells, including those that have little to no expression at standard potassium and phosphate concentrations (Figs. 1 and 2). Analysis of bmh2∆ SPL2-GFP cells showed that a smaller fraction of the cells lack expression of SPL2-GFP. Less effect of the *bmh2*∆ deletion compared to the *bmh1*∆ deletion may be expected as the levels of the Bmh2 protein are 5–10-fold lower than those of the Bmh1 protein [33].

Heterogenic expression of *PHO* genes has been reported before [31]. These authors identified positive and negative feedback loops, leading to bi-stability in phosphate transporter usage and as a result individual cells expressing predominantly either low- or high-affinity transporters. *SPL2* plays a key role in these feedback loops as induction of *SPL2* is necessary and sufficient for *PHO* pathway-dependent down-regulation of low-affinity transporters, causing individual cells to express either low- or high-affinity transporters. The origin of the cell-to-cell variability in gene expression in genetically identical cells is unclear. It may arise from noise in gene expression [31, 34–37]. Research on *PHO5* has provided evidence that nucleosome positioning plays a role in variations in gene expression at the single cell level [38, 39]. Under phosphate-rich conditions, *PHO5* gene expression is very low, and the promoter is occupied by nucleosomes. Upon phosphate starvation, there is a shift to a more nucleosome-free state. However, the small fraction of cells that expresses *PHO5* under phosphate-rich conditions also exhibits this nucleosome-free state [39]. Non-coding RNA may also be involved in the cell-to-cell variation in gene expression [40]. As 14-3-3 proteins have hundreds of binding partners 14-3-3 proteins can potentially influence transcription at different levels. 14-3-3 proteins bind to histone H3 [41] indicating that the effect may be directly at the nucleosome level. It has been shown in mammalian cells that interaction of 14-3-3 proteins with histone H3 leads to transcriptional activation [42]. On the other hand, after deletion of *PHO80* the *PHO84* and *SPL2* promoters are strongly activated, both in the wild type and *bmh1*∆ cells and the effect of the *bmh1* deletion is lost, suggesting that 14-3-3 proteins do not affect processes downstream of Pho4. It has been shown that Pho4 can induce changes in the nucleosome positioning of the *PHO5* gene [43]. Thus, 14-3-3 proteins may influence nucleosome positioning indirectly by affecting the activation state of Pho4. In addition to *PHO84* and *SPL2*, many more genes including *PHO5* are activated by Pho4 during phosphate depletion (Additional file 2). Except for affecting the activation state of Pho4, Bmh1 may also affect transcription of the *PHO* genes at other levels. Recently, it has been shown that in addition to Pho4 the Aft2 transcription factor is involved in the regulation of *SPL2* [44]. However, regulation of this transcription factor by 14-3-3 proteins still has to be shown.

The observation that the interaction between 14-3-3ζ and phosphorylated human HspB6 is destabilized at physiologically relevant phosphate concentrations (5–15 mM) [26], may be of interest. This observation may indicate that modulation of the interaction between 14-3-3 proteins and their interaction partners by phosphate contributes to the regulation of the expression of *PHO* genes. One of the relevant 14-3-3 binding partners is the Kcs1 protein [45–48], an inositol hexakisphosphate and inositol heptakisphosphate kinase [49]. The Pho80 – Pho85 complex is inhibited by Pho81 in conjunction with inositol heptakisphosphate (eIP7) [50]. The function of Kcs1 in phosphate regulation is unclear, but Kcs1 may have a negative effect on the production of eIP7 and may play a role in the establishment of feedback loops stabilizing the activation state of the *PHO* regulon [51]. However, the role of the interaction of Kcs1 with 14-3-3 proteins is unknown.

## Additional files

Additional file 1: Effect of deletion of *BMH1 or BMH2* on the RNA levels after growth in YNB with 50 or 0 mM KCl. (XLSX 1235 kb)

Additional file 2: Effect of *BMH1* and *BMH2* deletion and potassium starvation on RNA levels of *PHO* genes. (PDF 56 kb)

Additional file 3: Genes of which the RNA level increased or decreased significantly (*P* < 0.01) more than 2.0-fold upon deletion of *BMH1* or *BMH2* after growth in YNB with 50 mM KCl. (XLSX 13 kb)

Additional file 4: Localization of free GFP expressed under control of the *SPL2* promoter after cultivation in the absence of potassium. (PDF 84 kb)
