# Supplementary material for: Lack of 14-3-3 proteins in Saccharomyces cerevisiae results in cell-to-cell heterogeneity in the expression of Pho4-regulated genes SPL2 and PHO84
Source: BMC Genomics. 2017 Sep 6;18:701. doi: 10.1186/s12864-017-4105-8 (PMC5588707; doi:10.1186/s12864-017-4105-8)
Supplement: Supplementary file 2 — Effect of BMH1 and BMH2 deletion and potassium starvation on RNA levels of PHO genes. (PDF 56 kb) [file 12864_2017_4105_MOESM2_ESM.pdf]

Additional file 2. Effect of *BMH1* and *BMH2* deletion and potassium starvation on RNA levels of *PHO* genes.

	RNA level (reads per million) (± SD)					
**Gene[a]**	**BY4741**		**bmh1Δ**		**bmh2Δ**	
	**50 mM (n = 4)**	**0 mM (n = 4)**	**50 mM (n = 3)**	**0 mM (n = 3)**	**50 mM (n = 3)**	**0 mM (n = 3)**
PHO84	50 ± 27	516 ± 214	2 ± 1	243 ± 140	6 ± 0.5	235 ± 16
SPL2	31 ± 5	224 ± 69	3 ± 0	95 ± 33	7 ± 1	163 ± 26
GDE1	100 ± 68	108 ± 71	23 ± 4	51 ± 15	59 ± 5	77 ± 20
VTC2	260 ± 48	552 ± 79	137 ± 29	286 ± 29	220 ± 21	316 ± 35
PHO8	31 ± 15	260 ± 147	12 ± 3	59 ± 14	19 ± 4	118 ± 43
PHO5	4 ± 4	102 ± 58	11 ± 8	70 ± 63	0.8 ± 0.2	17 ± 8
PHO11	13 ± 13	114 ± 75	23 ± 20	81 ± 68	1.5 ± 0.5	12 ± 5
PHO12	0.1 ± 0.1	0.4 ± 0.6	0.03 ± 0.06	0.24 ± 0.07	0.1 ± 0.2	0.2 ± 0.3
PHO89	16 ± 19	29 ± 13	7 ± 4	9 ± 3	8 ± 2	9 ± 7
PHO86	221 ± 97	311 ± 31	232 ± 117	286 ± 51	206 ± 40	266 ± 72
PHO81	35 ± 21	138 ± 83	29 ± 17	60 ± 38	52 ± 9	156 ± 30
VTC3	107 ± 77	531 ± 297	24 ± 16	155 ± 83	26 ± 5	108 ± 15
VTC4	74 ± 37	257 ± 70	86 ± 52	214 ± 99	68 ± 24	193 ± 51
VTC1	713 ± 436	896 ± 291	251 ± 120	495 ± 148	220 ± 39	497 ± 22
HOR2	24 ± 16	27 ± 15	48 ± 11	46 ± 10	20 ± 5	63 ± 21
CTF19	0.9 ± 0.8	0.5 ± 0.6	0.6 ± 0.1	0.9 ± 0.1	0.6 ± 0.6	0.9 ± 0.6
HIS1	590 ± 311	333 ± 93	616 ± 338	492 ± 118	607 ± 161	236 ± 67
PPN1	238 ± 56	311 ± 71	187 ± 46	217 ± 75	315 ± 5	303 ± 40
PHM6	11 ± 6	66 ± 25	4 ± 2	21 ± 11	3 ± 1	19 ± 2
PHM7	4 ± 2	2 ± 2	7 ± 4	4 ± 2	3 ± 0.2	2 ± 1
PHM8	16 ± 9	82 ± 64	22 ± 10	80 ± 66	9 ± 3	50 ± 12
KRE29	1.0 ± 1.2	3 ± 3	0.3 ± 0.2	0.7 ± 0.4	0.2 ± 0.02	0.6 ± 0.5

a, The list of *PHO* gens was taken from:

Ogawa N, DeRisi J, Brown PO. New components of a system for phosphate accumulation and polyphosphate metabolism in *Saccharomyces cerevisiae* revealed by genomic expression analysis. Mol. Biol. Cell. 2000;11:4309–21.
