# Supplementary material for: Lack of 14-3-3 proteins in Saccharomyces cerevisiae results in cell-to-cell heterogeneity in the expression of Pho4-regulated genes SPL2 and PHO84
Source: BMC Genomics. 2017 Sep 6;18:701. doi: 10.1186/s12864-017-4105-8 (PMC5588707; doi:10.1186/s12864-017-4105-8)
Supplement: Supplementary file 4 — Localization of free GFP expressed under control of the SPL2 promoter after cultivation in the absence of potassium. (PDF 84 kb) [file 12864_2017_4105_MOESM4_ESM.pdf]

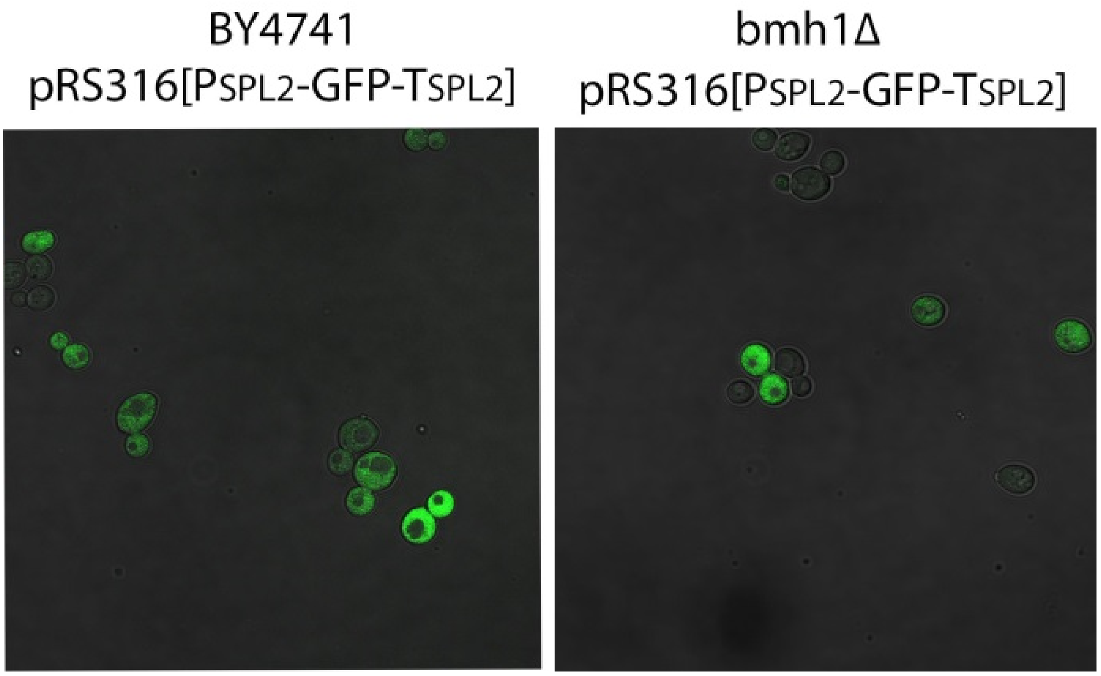

Additional file 4. Localization of free GFP expressed under control of the *SPL2* promoter after potassium starvation. BY4741 and Δbmh1 cells containing pRS316[P_{SPL2}-GFP-T_{SPL2}] were grown for 2 hrs in YNB medium without KCl.
